# Highly Efficient Perfect Vortex Beams Generation Based on All-Dielectric Metasurface for Ultraviolet Light

**DOI:** 10.3390/nano12193285

**Published:** 2022-09-21

**Authors:** Muhammad Danial Shafqat, Nasir Mahmood, Muhammad Zubair, Muhammad Qasim Mehmood, Yehia Massoud

**Affiliations:** Innovative Technologies Laboratories (ITL), King Abdullah University of Science and Technology, Thuwal 23955, Saudi Arabia

**Keywords:** ultraviolet, silicon nitride, perfect vortex beam, spiral phase plate, axicon

## Abstract

Featuring shorter wavelengths and high photon energy, ultraviolet (UV) light enables many exciting applications including photolithography, sensing, high-resolution imaging, and optical communication. The conventional methods of UV light manipulation through bulky optical components limit their integration in fast-growing on-chip systems. The advent of metasurfaces promised unprecedented control of electromagnetic waves from microwaves to visible spectrums. However, the availability of suitable and lossless dielectric material for the UV domain hindered the realization of highly efficient UV metasurfaces. Here, a bandgap-engineered silicon nitride (Si_3_N_4_) material is used as a best-suited candidate for all-dielectric highly efficient UV metasurfaces. To demonstrate the wavefront manipulation capability of the Si_3_N_4_ for the UV spectrum, we design and numerically simulate multiple all-dielectric metasurfaces for the perfect vortex beam generation by combing multiple phase profiles into a single device. For different numerical apertures (NA =0.3 and 0.7), it is concluded that the diffracted light from the metasurfaces with different topological charges results in an annular intensity profile with the same ring radius. It is believed that the presented Si_3_N_4_ materials and proposed design methodology for PV beam-generating metasurfaces will be applicable in various integrated optical and nanophotonic applications such as information processing, high-resolution spectroscopy, and on-chip optical communication.

## 1. Introduction

Ultraviolet (UV) radiation refers to invisible electromagnetic waves with the highest photon energy. The sun is one of the natural sources of UV radiation, whereas Johan W. Ritter discovered these radiations artificially in 1801 and observed a comparatively stronger interaction with silver chloride-soaked paper than the violet radiations. UV radiations play an indispensable role in numerous exciting applications that include imaging, microscopy, spectroscopy, quantum optics, micro-machining, light therapy, communication, and many more. For instance, circular dichroism spectroscopy exploiting deep-UV radiations is widely employed in the characterization and evaluation of different pharmaceuticals [1,2]. With the ability to cause ionization and breakage of chemical bonds, UV radiations are extensively employed in waste-water treatment, air purification, and virus inactivation [3,4]. So far, the manipulation of UV light mainly depends on a chain of traditional reflective or refractive optical components that results in bulky and expensive systems. Moreover, in contrast to the wide variety of suitable optical materials for near-infrared and visible spectra, the availability of cost-effective and appropriate materials for the UV regime is limited and requires challenging fabrication techniques. For example, lossless high-refractive-index dielectric materials are preferred for numerous optoelectronics and photonics application such as waveguides, filters, and antireflection coatings, etc. Most of the existing high-refractive-index materials possess a smaller bandgap, thus characterized as lossy materials for smaller wavelengths [5,6,7]. To meet the increasing demand for miniaturization and integration with nanophotonic technologies, the exploration of novel UV materials and associated fabrication techniques is a striking hotspot for the research community.

Unlike conventional optical components that control the wavefront of electromagnetic waves through their spatially varying shapes (propagation effect), artificially engineered nanostructures (metamaterials and metasurfaces) exhibit an unprecedented ability to tailor the wavefront of the indent light by judiciously devising their fundamental building blocks [8,9,10,11,12]. Metamaterials are defined as materials that have been engineered to demonstrate those properties which are not achievable through naturally existing materials [13,14,15,16]. A planar version of these three-dimensional metamaterials, dubbed metasurfaces, comprising spatially distributed nanoantennae, possesses an extraordinary ability to control the wavefront of incident light at the micron scale. This can be done by arranging the nanoantennae in arrays periodically by varying its size or rotation. These nanoscale unit cells can be intelligently engineered, and the resulting nanophotonic devices exhibit similar or even superior optical performance compared to their traditional bulk-optic counterpart, but with a significantly reduced size. Furthermore, nanophotonic technology emerges as a potential candidate for high-performance–low-loss optoelectronic systems exhibiting novel functionalities and smaller footprints [17,18,19,20,21,22]. Metasurfaces, the two-dimensional nanophotonic devices, consist of spatially distributed nanoantennae that could be designed to tailor the amplitude, phase, and polarization of the incident light by engineering the geometry and position of their building blocks [23,24,25]. The nanoscale thickness and planar footprints of metasurfaces enable their fabrication using single-step lithography technologies and allow them to take full advantage of mature semiconductor industries. So far, the research community has demonstrated numerous high-performance metasurfaces exhibiting a chain of functionalities including beam shaping [26], high-resolution imaging [27,28,29], meta-absorbers [30,31,32,33], asymmetric light transmission [34,35,36,37], hologram projection [38,39,40,41,42,43,44], and structured light generation [45,46,47,48,49,50,51,52,53,54].

The design principles of UV metasurfaces are quite similar to that of operating in the visible to microwave regimes, but strong material absorption and the high photon energy of UV light limit the realization of highly-efficient UV nanophotonic devices [55]. With the employment of large bandgap materials and the advancement of nano-fabrication technologies, recent years have witnessed a revolutionary development of miniaturized UV metasurfaces and their potential application in lithography [56], holography [55,57], information security [58], and many more sectors. Recently, hafnium oxide (HfO_2_) and niobium pentoxide (Nb_2_O_5_) have been employed to realize UV metasurfaces; however, meager throughput and the requirement of challenging fabrication processes hinder the applicability of these materials for nanophotonic devices [58,59,60]. Here, in this work, we utilized a bandgap-engineered silicon nitride (Si_3_N_4_) material whose extinction coefficient (*k*) is reduced by optimizing the SiH_4_:N_2_ gas ratio during deposition while maintaining the sufficient high refractive index (*n*) for the UV wavelengths. In contrast to available UV transparent materials, Si_3_N_4_ is compatible with well-matured complementary metal-oxide-semiconductor (CMOS) technology. The Si_3_N_4_ material can be deposited using plasma-enhanced chemical vapor deposition (PECVD) under different gas ratios of SiH_4_:N_2_. The engineered Si_3_N_4_ possesses a bandgap of ~5.9 eV and enables it to achieve 76.28% transmission efficiency for a design wavelength of 300 nm. As a proof of concept, we have designed and numerically simulated multiple UV metasurfaces that are capable of controlling the wavefront of incident light to generate perfect vortex (PV) beams with different numerical apertures. The proposed PV beam-generating metasurfaces consist of high-efficiency rectangular-shaped nanoantennae of Si_3_N_4_ sitting on the sapphire (Al_2_O_3_) substrate. The proposed dielectric materials and associated less-challenging fabrication techniques will be promising candidates for planar, cost-effective, and lightweight nanophotonic devices for the UV spectrum. Furthermore, the CMOS compatibility of Si_3_N_4_ will be an additional advantage.

## 2. Theory and Design

Electromagnetic waves possessing orbital angular momentum (OAM) promised a superior degree of freedom in light–matter interaction, which lies at the heart of theory and applications in modern physics. Contrary to photonic spin angular momentum (SAM) associated with the circularly polarized light, which is limited by two states, the AOM of the optical vortex (OV) beam exhibiting phase singularity in the center enables a new dimension with approximately unbounded states theoretically. In mathematics, singularity is a point where a function is undefined or takes an infinite value, so the OV beam exhibits zero intensity and corresponding undefined phase distribution in the center, forming a helical phase wavefront. The infinite number of eigenstates of the OAM enables support for an infinite number of data bits and promises a superior degree of freedom in information processing for optical communication. In 1992, Allen et al., theoretically construed the OV beams containing the azimuthal phase gradient term exp (jℓφ), where ℓ presents the topological charge and φ is the azimuthal angle [46]. Electromagnetic waves with helical phase distribution have been extensively applied in exciting applications including imaging and microscopy, optical manipulation [61], information processing [62], and data storage [63]. However, the radius of the doughnut-shaped intensity of the OV beam is highly dependent on its topological charge. To meet the increasing demand of high data transmission for optical communication, a serious bottleneck arises while coupling the OV beam of different topological charges into an optical fiber. Thus, it is highly desirable to control the ring radius of OV beams at will, not only for information processing, but also for other applications such as particle manipulation.

This aforementioned problem is addressed by introducing the concept of perfect vortex (PV) beams where the ring radius of the OV beam remains unaltered regardless of the value of the topological charge. The existing techniques of PV beam generation mostly exploit the Fourier transformation of the Bessel–Gaussian beam [64]. However, these techniques include a series of bulky refractive or reflective optical elements, which inevitably lead the overall optical system towards complexity and bulkiness and hinder the integration of PV beams with nanophotonic circuits. Recently, the demonstration of optical metasurfaces has opened up new avenues to tailor the wavefront of electromagnetic waves and enable remarkable freedom in integration and flexibility over traditional optical components. In this regard, Wu et al., generated the PV beams through metasurfaces by replacing the Fourier transform lens, axicon, and spiral phase plate with their planar counterparts (metasurfaces), respectively. Later on, Zhang et al., integrated the phase profiles of three constituent optical elements into a single plasmonic device to generate PV beams directly. However, most of the reported PV beam-generating metasurfaces operate well in the optical regime and suffer significant material absorption losses at shorter wavelengths in the ultraviolet regime. Here, we utilized a bandgap-engineered Si_3_N_4_ material as a best-suited candidate to demonstrate the all-dielectric highly efficient metasurface to generate PV beams in the UV spectrum. The designed metasurfaces consist of spatially distributed rectangular-shaped nanoantenna, working as a half-wave plate to control the wavefront of the incident light and generate the PV beam in the transmission mode. The numerical results indicate the descent transmission efficiency of 76.28% at the design wavelength of 300 nm.

For starting the approach toward the PV beam, we first take a special case of the OV beam, whose intensity is independent of the topological charge. Equation (1) represents a Dirac function for this OV beam:

(1) $$E\left(\rho ,\varphi \right)=\delta \left(\rho -\rho_{o}\right)\;\exp\left(j\ell \varphi \right)$$

The polar coordinates are (ρ,φ), ℓ is the topological charge, and the radius of the ring is $\rho_{o}$. There is a specific problem with Equation (1), as it cannot be implemented experimentally in a real-time situation. Therefore, Equation (2) is required to approximate the experiential model of the OV beam independent of the topological charge. Δρ is the smaller width used for the approximation:

(2) $$E\left(\rho ,\varphi \right)=\exp\left[-\frac{{\left(\rho -\rho_{o}\right)}^{2}}{\Delta \rho^{2}}\right]\exp\left(j\ell \varphi \right)$$

In the mathematical theory, the PV beam will be attained after the Fourier transformation of the Bessel beam in the ideal state. But for a real-world resemblance, a good way is to take the transformation of the Bessel–Gaussian (BG) function. The BG beam is represented in a mathematical Equation (3):

(3) $$E_{BG}\left(\rho ,\varphi \right)=J_{\ell }\left(k_{r}\rho \right)\exp\left(j\ell \varphi \right)\exp\left[-\frac{\rho^{2}}{w_{g}{}^{2}}\right]$$

And we will take the transform of the Equation (3), which will give us the result Equation (4):

(4) $$E\left(\Upsilon ,\Theta \right)=\iota^{\ell -1}\frac{w_{g}}{w_{o}}\exp\left(j\ell \varphi \right)\exp\left(-\frac{\Upsilon^{2}+\Upsilon_{r}{}^{2}}{w_{o}{}^{2}}\right)I_{\ell }\left(\frac{2{\Upsilon \Upsilon }_{r}}{w_{o}{}^{2}}\right)$$

(5) $$w_{o}=\frac{2f}{kw_{g}}\;,\;k=\sqrt{k_{r}{}^{2}+k_{z}{}^{2}}$$

The $w_{g}$ is the radius of the beam that will fix the total field, and $w_{o}$ is the radius of the Gaussian beam with focal length *f*. $I_{\ell }$ is a Bessel function of ℓth order. As this is a Bessel–Gaussian beam, both Bessel and Gaussian functions will add up to make the final amplitude. This will give rise to a vortex beam with a constant ring size. The radius of the beam will be dependent upon the constitution values of the axicon. The intensity of the PV beam is expressed as

(6) $$I_{PV}\left(\Upsilon ,\Theta \right)=\frac{w_{g}{}^{2}}{w_{o}{}^{2}}\exp\left(-\frac{2{\left(\Upsilon -\Upsilon_{r}\right)}^{2}}{w_{g}{}^{2}}\right)$$

The intensity of the PV beam is not dependent on the topological charge, so the ring radius will remain constant. All the above discussion tells us that the PV beam will be generated using a spiral plate (to introduce the topological charge), an axicon (to generate the BG beam), and a lens (for Fourier transformation). Most of our math is inspired by [2]. We encode the phase profile of all the above things on a single metasurface with the help of the PB Phase, which will be expressed as

(7) $$\varphi_{PV}\left(x,y\right)=\varphi_{spiral}\left(x,y\right)+\varphi_{lens}\left(x,y\right)+\varphi_{axicon}\left(x,y\right)$$

(8) $$\varphi_{spiral}\left(x,y\right)=\ell \;\cdot tan^{-1}\left(\frac{y}{x}\right)$$

(9) $$\varphi_{lens}\left(x,y\right)=\frac{2\pi }{\lambda_{d}}\left[\sqrt{\left(x^{2}+y^{2}\right)+f^{2}}-f\right]$$

(10) $$\varphi_{axicon}\left(x,y\right)=\frac{2\pi }{\lambda_{d}}\;\cdot \sqrt{\left(x^{2}+y^{2}\right)}\;\cdot \;NA$$

where x and y are rectangular coordinates for unit cells. Equation (8) represents a spiral plate phase profile that is used to insert the topological charge (ℓ). Equation (9) defines a lens with the focal length f, which is used for the Fourier transformation. Equation (10) introduces an axicon phase profile with numerical aperture *NA*. Here $\lambda_{d}$ is the designed wavelength.

**Figure 1:**
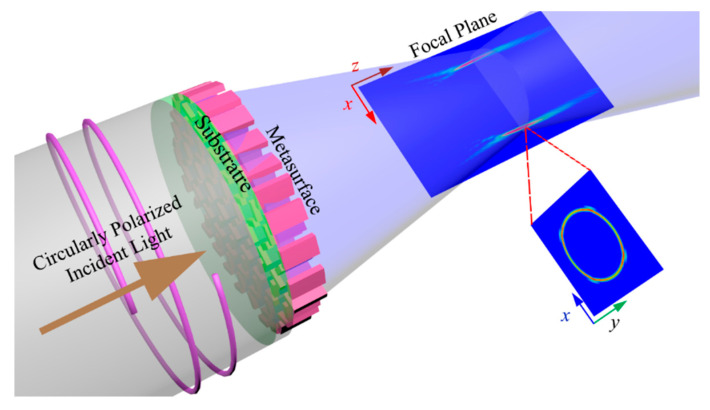
Working principle of silicon nitride-based perfect vortex beam-generating metasurfaces. The phase profiles of the spiral phase plate, lens, and axicon are combined and encoded to address the placement and in-plane rotation of the high-efficiency nanoantennae. Ultraviolet light with $\lambda_{d}=300\;\mathrm{nm}$ impinges from the substrate side, and the diffracted light from the metasurface is captured on the outside.

The working principle of the PV beam-generating metasurface is illustrated in Figure 1, where the circularly polarized light of 300 nm wavelength is impinged on the designed structure from the substrate side.

**Figure 2:**
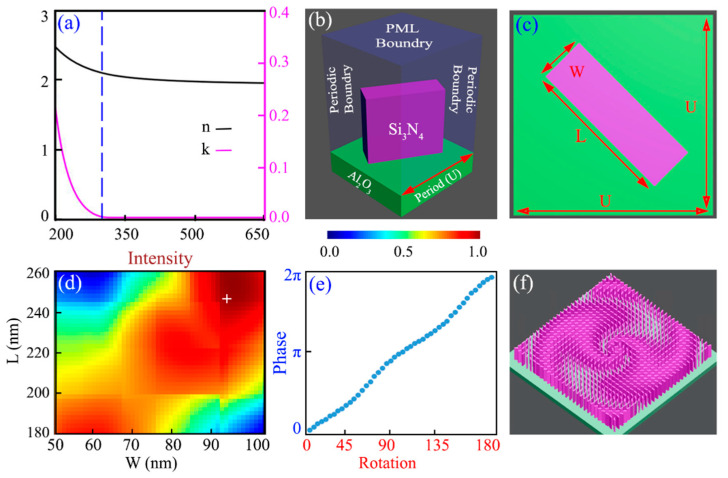
Material properties and parametric optimization of the unit cell. (**a**) Ellipsometry data of the engineered silicon nitride material deposited using plasma-enhanced chemical vapor deposition (PECVD) under different gas ratios of SiH4:N2. Presented dielectric material exhibits negligible absorption loss k=0.00011 at the ultraviolet wavelengths. (**b**) Schematic illustration of the simulated fundamental building block where the rectangular-shaped nanoantenna of Si_3_N_4_ sitting on the sapphire substrate is used for parametric analysis. Finite difference time domain-based FDTD Solution software is used to perform the optimization, where periodic boundary conditions are applied along the x- and y-axes, and PML boundaries are applied along the *z*-axis. (**c**) The top view of the unit cell consists of a rectangular bar of Si_3_N_4_ of the sapphire substrate. Here, U presents the period of the unit cell, L and W indicates the length and width of the nanoantenna, respectively. Numerical optimization of the unit cell reveals the optimized values as U=280 nm, L=240 nm, and W=92 nm. (**d**) The numerically simulated transmission intensity distribution of the diffracted cross-polarized light for wavelength $\lambda_{d}=300\;\mathrm{nm}$. To obtain the optimized value of the nanoantenna, its length and width are swept in the software. The “+” sign on the (**d**) plot indicates the selected dimensions of the nanoantenna as mentioned above. (**e**) Verification of the nanoantenna to act as a halfwave plate. Nanoantenna is in-plane rotated and the diffracted with the opposite handedness is received and analyzed for complete phase coverage. (**f**) Schematic diagram of the designed UV metasurface with topological charge n=2 for PV beam generation.

To demonstrate the wavefront manipulation capability of the Si_3_N_4_ for UV light, we design multiple PV beam-generating metasurfaces with different topological charges and numerical apertures. The fundamental building block of these metasurfaces is an asymmetric nanoantenna of Si_3_N_4_ material sitting on the sapphire substrate that works according to the geometric phase scheme. Figure 2a describes the ellipsometry (*n* and *k*) data of the engineered Si_3_N_4_ material where the vertical dashed line indicates the values of n=2.069 and k=0.00011 for $\lambda_{d}=300\;\mathrm{nm}$. With the fixed value of the refractive index of the deposited Si_3_N_4_ material, a sufficient height of the nanoantenna is required for the complete (0–2π) phase coverage. This can be understood with the help of Equation (12):

(11) $$\varphi =\frac{2\pi }{\lambda_{d}}\cdot h\cdot \varepsilon_{eff}$$

where $\lambda_{d}$ describes the design wavelength (300 nm in our case), h is the height of the nanoantenna, and $\varepsilon_{eff}$ represents the effective refractive index. According to the indexed waveguide theory [25], this effective refractive index can be described as a combination of air (or other material) and targeted material media within a specific region. Its value varies between the refractive indices of both the materials. Therefore, to cover the complete phase distribution and to fulfill the fabrication requirements (aspect ratio and spacing between neighboring antennae), a 500 nm height of the nanoantenna is chosen. However, in case of the availability of very good fabrication techniques (e.g., atomic layer deposition), the height of the nanopillar can be chosen ranging from 400 nm to 500 nm. The sufficient high refractive index and negligible absorption loss of the Si_3_N_4_ material for the UV domain make it the best-suited candidate for highly efficient all-dielectric transmissive metasurfaces.

Commercially available finite difference time domain-based Ansys Lumerical Inc. FDTD Solution 2022 R1.4, USA is used as a numerical solver to perform the numerical simulations of the unit cell and complete metasurfaces. The 500 nm tall rectangular-shaped building block is numerically optimized to work as a halfwave plate which is capable of achieving the complete (0–2π) phase coverage for the design wavelength. The period of the unit cell is selected in such a way that it allows the constructive interference of the diffracted light and exhibits a maximum possible transmission efficiency of 76.28%. Here, the term “transmission” implies that the metasurfaces are working in transmission mode. The input light has impinged on the metasurface from the substrate side, and the diffracted light is captured after transmission from the metasurfaces. With a 280 nm period along the x- and *y*-axis, the unit cell consists of an optimized nanoantenna having L=240 nm and W=92 nm. A schematic description of the simulated unit cell in the Ansys Lumerical Inc. FDTD Solution 2022 R1.4, USA is presented in Figure 2b, where periodic boundary conditions are used along the x- and *y*-axis and perfect matching layer (PML) boundaries are applied along the *z*-axis. During simulations, the “auto non-uniform” mesh type is used with a minimum mesh step size of 0.25 nm, and the minimum auto-shutoff level was set to $1\times 10^{-5}$. For the case of unit cell optimization, the air region above the nanoantenna was taken as 15λ, whereas for the case of complete metasurfaces, the air region was taken as double the defined focal plane. Initially, the period of the unit cell is optimized by taking the cylindrical nanoantenna. This technique significantly decreases the computation requirements, as only a single parameter—the radius of the cylindrical nanoantenna—is to be swept against the period. From the simulated profile of transmission intensity for radius vs. period, an initial value of the period of the unit cell is taken, which ensures the maximum possible transmission efficiency. In the next step, the cylindrical nanoantenna is replaced with a rectangular bar as presented in Figure 2c. At this stage, with a fixed period of the unit cell, the length and width of the rectangular nanoantenna are swept from (60–250 nm). A UV light with left-hand circularly polarized (LCP) having a 300 nm wavelength impinges from the substrate side, and the diffracted light is captured by the XY-monitor placed about more than 10λ away from the structure. At the output side, diffracted light with opposite handedness is captured and analyzed to obtain the physical dimensions of the nanoantenna.

The cross-polarized transmission efficiency of the diffracted light is illustrated in Figure 2d, which enables the selection of the optimum values of length and width of the nanoantenna for a maximum possible conversion efficiency of 76.28%. The Pancharatnam–Berry (PB)-based phase-achieving mechanism is extensively demonstrated where the anisotropic nanoantenna is illuminated with circularly polarized light to get the maximum possible cross-polarized light at the output side. The amplitude of the diffracted complex field can be represented as below [64]:

(12) $$E_{d}\left(\theta \right)=\frac{t_{o}+t_{e}}{2}E_{rcp}+\frac{t_{o}-t_{e}}{2}E_{lcp}\cdot e^{j2\theta }$$

where $t_{o}$ and $t_{e}$ indicate the coefficients of forward scattered light along the two principle axes of the nanoantenna, respectively. $E_{rcp}$ and $E_{lcp}$ represent the amplitude of the co- and cross-polarized light. The first term on the right hand side of the Equation (12) indicates the co-polarized light with the same handedness, and the second term represents the cross-polarized light having a different handedness from the incident light. It is apparent that the cross-polarized light component of the diffracted field contains an additional phase of 2θ, where θ indicates the in-plane rotation angle of the optimized nanoantenna. It is concluded that the latter term provides the complete (0–2π) phase coverage by simply rotating the nanoantenna from 0 to 180°. To further ensure the working of a completely optimized unit cell as a halfwave plate, numerical simulation is performed to obtain the complete (0–2π) phase coverage vs. the rotation of the nanoantenna. Figure 2e demonstrates the rotation vs. phase profile of the optimized nanoantenna, which shows excellent agreement with the expected result. With the in-plane rotational of the nanoantenna from $0–180^{{}^{\circ}}$, the complete (0–2π) phase distribution of the cross-polarized light is achieved.

## 3. Results and Discussion

The schematic of the proposed metasurface is presented in Figure 2f, where Equation (7) is used to address the placement and in-plane rotation of the Si_3_N_4_ nanoantennae. As discussed in the previous section, the PV beams can be generated by combining the spiral phase plate, Fourier lens, and axicon; the same design strategy here merges the phase profiles of these three optical elements. Equations (8)–(10) describe the phase profiles of the spiral phase plate, lens, and axicon, respectively. In contrast to conventional optical systems, we encoded the phase profiles of all the required components in a single-layered metasurface ensuring ultracompact systems. To be specific, the proposed metasurfaces are designed with the numerical aperture NA=0.3 and 0.7. The value of the numerical aperture dictates the focal plane of the converging light. The position of the focal plane is inversely proportional to the numerical aperture. As the numerical aperture increases (or approaches unity), the focal plane gets closer to the surface of the device, and, as a result, the light bending gets tough. In conventional bulk optics, one cannot obtain a numerical aperture greater than 0.707. Here, the purpose of the demonstration of different numerical apertures (e.g., NA=0.3 and 0.7) is to demonstrate the ability of the proposed metasurfaces to efficiently control the wavefront of the incident light. Furthermore, each category is simulated with topological charge ℓ=2, 4,and 6. All the designed metasurfaces with a 30 µm×30 µm size are numerically simulated with PML boundary conditions on each side. The focal length of the constituent lens is chosen in such a way as to obtain the approximate non-diffracting behavior of the PV beams. For this purpose, with a 30 μm length of the metasurface and maintaining the NA=0.3, the focal length of the lens is decreased by 1/3 times the actual focal length. Similarly, for NA=0.7, the focal length is lowered 2/7 times the actual focal point. The highly efficient identical nanoantennae are spatially distributed on the sapphire substrate with rotation angle $\theta \left(x,y\right)=\varphi_{PV}\left(x,y\right)/2$, as described in Figure 2e.

**Figure 3:**
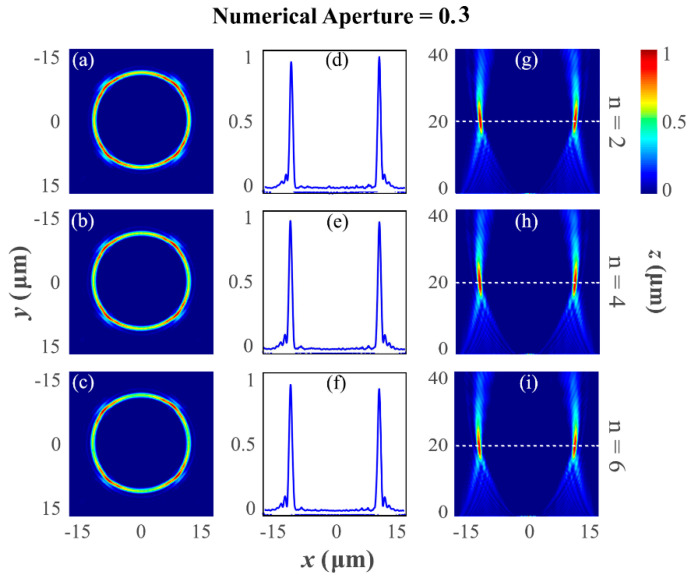
Far-field intensity distribution of the generated PV beams for numerical aperture NA=0.3 and different topological charges ℓ=2, 4, and 6 for the UV wavelength $\lambda_{d}=300\;\mathrm{nm}$. (**a**–**c**) The transverse intensity distribution of the diffracted light at the designed focal plane is ~20 μm (XY-monitor). It is seen that as the value of the topological charge increases, there is no variation in the ring size, thus verifying the generation of a perfect vortex beam. (**d**–**f**) indicate the full-width half-maximum of the light at the focal plane. While minting the numerical aperture NA=0.3, the focal length of the lens is decided as 1/3 times lower than the actual focal length. (**g**–**i**) The required non-diffracting type field behavior and formation of constant ring radius with maximum intensity at the desired focal plane.

Figure 3 describes the numerically simulated result of the PV beam-generating metasurfaces for numerical aperture NA=0.3. These PV beam-generating metasurfaces are designed and simulated with different topological charges, ℓ=2, 4, and 6. Most importantly, it is apparent that despite the value of the topological charge of the spiral phase plate, the ring radius of annular intensity remains almost unaltered. Figure 3a–c describes the far-field intensity distribution patterns captured by the XY-monitor placed at the focal length of 20 µm for topological charge ℓ=2, 4, and 6, respectively. Figure 3d–f illustrates the full-width half-maximum of the PV beam at the focal plane. To describe the evaluation process of the perfect vortex beams, the longitudinal intensity profile of the diffracted light is calculated and presented in Figure 3g–i, which is captured by the XZ-monitor. It is verified that the designed metasurfaces present non-diffracted-like perfect vortex beams, whose ring radii are independent of the embedded topological charge. It is verified that metasurfaces consisting of nanoscale resonators of Si_3_N_4_ material are capable of completely structuring the UV light by generating the PV beams.

**Figure 4:**
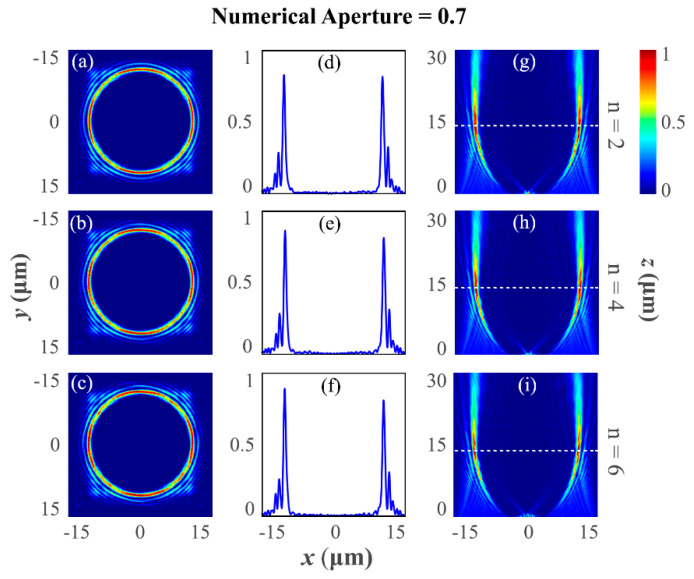
Far-field intensity distribution of the PV beam-generating metasurfaces for numerical aperture NA=0.7 and different topological charges ℓ=2, 4, and 6. (**a**–**c**) Transverse intensity distribution of the diffracted light at the designed focal plane at ~14 µm (XY-monitor). It is seen that regardless of the value of the topological charge, the ring radius is unaltered, thus verifying the generation of a perfect vortex beam. (**d**–**f**) indicate the full-width half-maximum of the light at the focal plane. While minting the numerical aperture NA=0.7, the focal length of the lens is decided as 2/7 times lower than the actual focal length. (**g**–**i**) The required longitudinal intensity distribution forms non-diffracting type field behavior and formation of constant ring radius with maximum intensity at the desired focal plane.

The numerically simulated results of the PV beam-generating metasurfaces for numerical aperture NA=0.7 are presented in Figure 4. As described earlier, the focal length of the lens is set in a way that it is 2/7 times less than the actual focal length (which is according to the NA=0.7). Figure 4a–c illustrates the transverse intensity profile of the diffracted light, which is captured by the XY-monitor at the defined focal plane. Similarly, Figure 4d–f presents the full-width half-maximum of the PV beam at the focal plane, and Figure 4g–i describes the longitudinal intensity profile of the diffracted light. It is observed that for a higher numerical aperture NA=0.7, the focusing point of the PV beam comes closer at ~15 µm. It is observed that the proposed metasurface design methodology for PV beam generation is verified for a shorter wavelength $\left(\lambda_{d}=300\;\mathrm{nm}\right)$.

**Figure 5:**
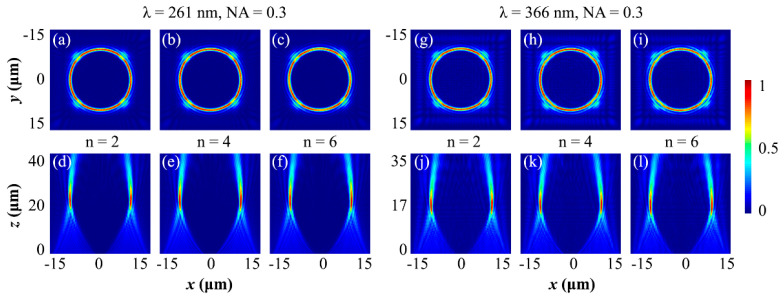
Numerically simulated behavior of the designed metasurfaces for other UV wavelengths. (**a**–**c**) and (**g**–**i**) presents the intensity distribution at the focal planes (XY planes) whereas (**d**–**f**) and (**j**–**l**) illustrates the results captured by the XZ monitors, placed along the propagation direction of the light. The designed metasurfaces are simulated for wavelengths λ=261 and 366 nm and concluded that the proposed Si_3_N_4_-based metasurfaces are capable of tailoring the complete wavefront of the incident light of different UV wavelengths.

It is well-known that the UV region covers the 100–400 nm wavelength range, and to verify the functionality of the proposed Si_3_N_4_-based metasurface for the rest of the UV spectrum, we further numerically simulated the designed metasurfaces for two other wavelengths, λ=261 and 366 nm. The numerically simulated results are presented in Figure 5, where the left-hand side of the figure illustrates the results for wavelength λ=261 nm, while the right-hand side indicates the simulated results for λ=366 nm. Figure 5a–c and Figure 5g–i represent the transverse intensity profile (XY-plane) of the diffracted light at the focal plane that validates the topological charge-insensitive ring radius of the generated PV beams for 261 nm and 366 nm wavelengths, respectively. Similarly, Figure 5d–f and Figure 5j–l illustrates the intensity distribution of the diffracted light along the propagation axis (XZ-plane). According to the dispersive nature of the presented dielectric material, Figure 5d–f and Figure 5j–l possesses different focal planes for different wavelengths of incident light. For both the UV wavelengths (261 nm and 366 nm), the numerically simulated results in Figure 5 exhibit the excellent ability of the proposed metasurfaces to completely tailor the incident light. It is verified that the proposed bandgap-engineered Si_3_N_4_ material is capable of controlling the wavefront of the other incident wavelength in the UV regime. Hence, it is safely concluded that the proposed Si_3_N_4_ material can prove itself as a best-suited candidate for the UV regime and can be successfully applied to different on-chip devices.

## 4. Conclusions

In conclusion, in utilizing the bandgap-engineered silicon nitride material for ultraviolet spectrum, we have demonstrated full control over the wavefront of electromagnetic waves through high-efficiency nanoantennae acting as half-wave plates. For proof of the concept, perfect optical vortex beams are generated by combining the phase profiles of the spiral phase plate, lens, and axicon that are encoded into a single-layered metasurface. The Ansys Lumerical Inc. FDTD Solution 2022 R1.4, USA was used to optimize the unit cell and to perform the numerical simulations of the designed metasurfaces. Silicon nitride-based all-dielectric metasurfaces exhibit a decent conversion efficiency of 76.28% for UV wavelength $\left(\lambda_{d}=300\;\mathrm{nm}\right)$. Moreover, perfect vortex beams-generating metasurfaces are designed for different numerical apertures (NA=0.3 and 0.7) and different topological charges, ℓ=2, 4, and, 6. We have achieved a non-diffracting type perfect vortex beam behavior of the diffracted light whose annular intensity ring radius remains insensitive to the topological charge. The proposed silicon nitride material is naturally compatible with well-matured complementary metal-oxide semiconductor technology, making it practically the best-suited candidate for cost-effective and highly efficient nanophotonic devices. We believe that the presented bandgap-engineered silicon nitride material and the associated perfect vortex beam-generating metasurfaces can find prominent applications in the UV domain.

## Data Availability

All the relevant data are presented in this research article but may be obtained from the authors upon reasonable request.
